# IL-37 Gene Modification Enhances the Protective Effects of Mesenchymal Stromal Cells on Intestinal Ischemia Reperfusion Injury

**DOI:** 10.1155/2020/8883636

**Published:** 2020-08-07

**Authors:** Dejun Kong, Yonghao Hu, Xiang Li, Dingding Yu, Hongyue Li, Yiming Zhao, Yafei Qin, Wang Jin, Baoren Zhang, Bo Wang, Hongda Wang, Guangming Li, Hao Wang

**Affiliations:** ^1^Department of General Surgery, Tianjin Medical University General Hospital, Tianjin, China; ^2^Tianjin General Surgery Institute, Tianjin, China; ^3^Department of Paediatric Surgery, Tianjin Medical University General Hospital, Tianjin, China

## Abstract

**Background:**

Ischemia reperfusion injury (IRI) is the major cause of intestinal damage in clinic. Although either mesenchymal stromal cells (MSCs) or interleukin 37 (IL-37) shows some beneficial roles to ameliorate IRI, their effects are limited. In this study, the preventative effects of IL-37 gene-modified MSCs (IL-37-MSCs) on intestinal IRI are investigated.

**Methods:**

Intestinal IRI model was established by occluding the superior mesenteric artery for 30 minutes and then reperfused for 72 hours in rats. Forty adult male Sprague-Dawley rats were randomly divided into the sham control, IL-37-MSC-treated, MSC-treated, recombinant IL-37- (rIL-37-) treated, and untreated groups. Intestinal damage was assessed by H&E staining. The levels of gut barrier function factors (diamine oxidase and D-Lactate) and inflammation cytokine IL-1*β* were assayed using ELISA. The synthesis of tissue damage-related NLRP3 inflammasome and downstream cascade reactions including cleaved caspase-1, IL-1*β*, and IL-18 was detected by western blot. The mRNA levels of proinflammatory mediators IL-6 and TNF-*α*, which are downstream of IL-1*β* and IL-18, were determined by qPCR. Data were analyzed by one-way analysis of variance (ANOVA) after the normality test and followed by post hoc analysis with the least significant difference (LSD) test.

**Results:**

IL-37-MSCs were able to migrate to the damaged tissue and significantly inhibit intestinal IRI. As compared with MSCs or the rIL-37 monotherapy group, IL-37-MSC treatment both improved gut barrier function and decreased local and systemic inflammation cytokine IL-1*β* level in IRI rats. In addition, tissue damage-related NLRP3 and downstream targets (cleaved caspase-1, IL-1*β*, and IL-18) were significantly decreased in IRI rats treated with IL-37-MSCs. Furthermore, IL-1*β*- and IL-18-related proinflammatory mediator IL-6 and TNF-*α* mRNA expressions were all significantly decreased in IRI rats treated with IL-37-MSCs.

**Conclusion:**

The results suggest that IL-37 gene modification significantly enhances the protective effects of MSCs against intestinal IRI. In addition, NLRP3-related signaling pathways could be associated with IL-37-MSC-mediated protection.

## 1. Introduction

Intestinal ischemia reperfusion injury (IRI), one of the major causes of clinical acute intestine necrosis, is a life-threatening disease with high mortality and disability [1]. Intestinal IRI is common in multiple diseases including vascular occlusion, hemorrhagic shock, trauma, and small bowel transplantation [2, 3]. However, currently, there is no effective treatment available besides surgical intervention, and thus, a novel treatment is urgently needed [4].

The underlying mechanisms of intestinal IRI are complicated, and diverse factors are involved in the process [1]. Briefly, intestinal IRI is often correlated with circulatory diseases in the gastrointestinal system involving the superior mesenteric artery (SMA), weak innate immunity, weak adaptive immunity, and high levels of inflammation. At the early stage of ischemia, the endothelial barrier is destroyed resulting in an increase in vascular permeability. Simultaneously, reactive oxygen species (ROS) can be excessively produced. After ROS production, reperfusion upregulates the release of ROS, consequently disrupting normal ATP generation. Excessive ROS also cause severe oxidative stress, which can promote DNA damage, endothelial dysfunction, and local inflammatory responses. On the basis of ischemia, reperfusion triggers the release of intracellular and extracellular damage-associated molecular pattern molecules (DAMPs), resulting in the accumulation of inflammatory cells such as monocytes and dendritic cells [5].

The NOD-like receptor protein 3 (NLRP3) is a member of pattern recognition receptors and plays a key role in inflammatory responses via formation of an intracellular multiprotein complex known as NLRP3 inflammasome, which is the best characterized of all other inflammasomes [6]. Inflammasomes are important signal platforms in detecting sterile stressors and pathogenic microorganisms such as some DAMPs, which activate and release the highly proinflammatory cytokines interleukin 1*β* (IL-1*β*) and interleukin 18 (IL-18). In this process, ROS, as a kind of DAMPs, was excessively produced during IRI and then promotes NLRP3 inflammasome activation [7], subsequently assisting conversion of pro-caspase-1 into cleaved caspase-1; the activation of caspase-1 can upregulate the production and secretion of the cytokines such as IL-1*β* and IL-18 by cleavage of pro-IL-1*β* and pro-IL-18. At the same time, activation of inflammasome-associated inflammatory caspase-1 drives cleavage of the propyroptosis factor, gasdermin D, generating an N-terminal fragment that oligomerizes to form pores on the cell membrane and causes programmed cell death known as pyroptosis [8–11]. In previous studies, NLRP3 can be activated by different mediators such as DAMPs and/or PAMPs *in vivo*, and NLRP3 plays a crucial role in the development of IRI in various organs [7, 12, 13]. Therefore, inhibiting NLRP3 inflammasome activation may play a protective role in IRI.

Mesenchymal stromal cells (MSCs) are a group of stem cells derived from the embryonic mesoderm, which can differentiate into various kinds of cells [4, 14, 15]. MSCs have the proliferative, pluripotent, and immunomodulatory potentials that can help repair damaged tissues and improve tissue microenvironment [16]. Accumulating evidence has demonstrated that MSC treatment could alleviate IRI in various organs by inhibiting intensive inflammation, apoptosis, generations of oxidative stress, ROS, and immune overreactivity [17, 18].

Interleukin 37 (IL-37) is a novel cytokine that recently characterized a member in the IL-1 family, which plays a key role in limiting excessive and runaway inflammatory responses via suppressing both innate and adaptive immunity [19–21]. It has been demonstrated that a knockdown of endogenous IL-37 in human peripheral blood mononuclear cells results in increased production of several proinflammatory cytokines [22]. Human IL-37 transgenic mice are protected against metabolic syndrome, systemic inflammation reaction, DSS-induced colitis, and acute myocardial infarction. As an immunomodulatory factor, IL-37 has been tested in IRI models such as myocardial and renal IRI [23, 24].

Although MSCs and IL-37 could protect organs against IRI, their roles are limited for many reasons [25, 26]. Thus, it is necessary to combine them together to enhance their individual therapeutic effects. In this study, we successfully conducted IL-37 gene-modified MSCs and investigated their combined effects on the prevention of intestinal IRI and explored potential mechanisms of prevention.

## 2. Materials and Methods

### 2.1. Animals

Male Sprague-Dawley (SD) rats weighing 250-300 g were purchased from China National Institute for Food and Drug Control and were placed in a standard temperature environment, provided a standard diet, and provided water in the Animal Care Facility of Tianjin General Surgery Institute. All animal experimental operations were approved by the Institute of Animal Care and Use Committee at Tianjin Medical University and performed in accordance with the Guide for the Care and Use of Laboratory Animals.

### 2.2. Isolation and Culture of MSCs

MSCs were prepared according to the protocol described previously [4]. Briefly, in order to harvest the adipose tissue surrounding the inguen, rats were sacrificed and soaked in 75% alcohol for 10 min. Then, 200-300 *μ*L sterile PBS was added to every 0.5 g adipose tissue to prevent dehydration. The tissue was cut into <1 mm^3^ pieces, followed by the addition of type I collagenase solution (1 mg/mL, Solarbio, Beijing, China). The resulting tissue solution was placed in tubes which were subsequently placed on a shaker and incubated with constant agitation for 60 minutes (37°C, 200 rpm). Next, an equal volume of serum-containing medium was added to terminate digestion of the tissue. After that, the solution was centrifuged (1800 rpm, 5 min), the supernatant was discarded, and the remaining cells were washed twice with PBS. Lastly, cells were inoculated in a 15% fetal bovine serum (FBS, Hyclone, Tauranga, New Zealand) containing *α*-MEM medium (Hyclone, Tauranga, New Zealand) and 1% penicillin/streptomycin (Solarbio, Beijing, China) and subcultured after 7-10 days. MSCs were identified through detection of the cell's morphology and the cells' surface markers.

### 2.3. Preparation and Identification of IL-37-MSCs

The construction of the vector expressing IL-37 (NM_014439, IL-37 isoform b, which is best characterized, Ubi-MCS-3FLAG-SV40-EGFP-IRES-puromycin vector was used in this study, Ubi and SV40 here are promoters, FLAG and EGFP are marker genes, and MCS is a multiple cloning site), the required sequencing (results are shown in the supplement), and the lentiviral packaging were supported by GeneChem Inc., Shanghai, China. We conducted lentiviral transfection of MSCs according to the protocol provided by GeneChem Inc. (the vector lacking IL-37 was used as control). Lentiviral transfection is done with a suitable multiplicity of infection (MOI = 200, ratio of lentivirus to cell number) inside a biological safety cabinet. The cells then were observed under an inverted fluorescence microscope at 72 hours posttransfection, and IL-37 expression was identified by immunofluorescence (IF, details are shown in methods of immunofluorescence).

Furthermore, IL-37-MSCs were subjected to drug screening using a 1 *μ*g/mL puromycin (Solarbio, Beijing, China) solution to obtain high-purity IL-37-MSCs. Flow cytometry was performed on the resulting IL-37-MSCs before treatment to ensure cell purity and quality.

### 2.4. Flow Cytometry Analysis

The positive rate for markers of MSCs and IL-37-MSCs was analyzed by using flow cytometric analysis. In brief, MSCs were stained with fluorescent antibodies, including anti-CD29-FITC, anti-CD45-PE, anti-CD79a-PE, and anti-CD90-FITC (*e*Bioscience, San Diego, USA), according to the manufacturer's instruction. The percentages of various markers of MSCs were analyzed using the FlowJo software.

### 2.5. Immunofluorescence

We used immunofluorescence (IF) technology to identify IL-37 expression in IL-37-MSCs to ensure successful transfection; MSCs (negative control with lentiviral transfection which express GFP while lacking IL-37) were used as control. Briefly, first of all, MSCs were cultured on the slides pretreated with polylysine overnight. Then, cells were treated with 4% paraformaldehyde (PFA) for 30 minutes and subsequently treated with 0.1% Triton-X (Solarbio, Beijing, China) for 2 minutes. After that, cells were treated with 5% BSA for 30 minutes to reduce nonspecific antibody binding. Next, anti-IL-37 antibody (dilution at 1 : 250, Abcam, Cambridge, UK) was added to the slides for the night; after being washed three times by PBS, the cy3-conjugated goat anti-rabbit secondary antibody was added for half an hour and washed three times with PBS again. DAPI (Thermo Fisher Scientific, Waltham, USA) was added dropwise before the coverslip was placed over the slides. Lastly, the slides were observed under a fluorescence microscope.

### 2.6. Experimental Groups

To test the effects of IL-37-MSCs on protecting against intestinal IRI, SD rats were randomly assigned to five groups (*n* = 8 each group): sham control group, IL-37-MSC-treated IRI group, MSC-treated IRI group, recombinant IL-37- (rIL-37-) treated (PeproTech, New Jersey, US) IRI group, and untreated IRI group. In the beginning, rats got intraperitoneal injection of pentobarbital sodium (50 mg/kg), heating pad was used to maintain their temperature, and abdomen hair was removed. After abdominal disinfection, SMA was isolated by a midline incision into the abdominal cavity. In the sham control group, rats were only operated by opening and closing the abdomen, without clipping the SMA. In other groups, the roof of SMA was clipped for 30 min then recovered reperfusion. After restoring the blood supply and closing the incision, 2 × 10^6^ IL-37-MSCs, 2 × 10^6^ MSCs, 2 *μ*g rIL-37 (based on efficacy assessments from our preexperimental results, 2 *μ*g each rat was used in this study), and 1 mL PBS were separately injected into tail veins of the SD rats. Rats were maintained by continuous monitoring with a temperature-controlled self-regulated heating system after operation. After reperfusion for 72 hours, all rats were sacrificed for IRI assessment.

### 2.7. Tracing of Infused IL-37-MSCs

As IL-37-MSCs express GFP protein, we detected fluorescent protein expression in the damaged tissue to identify whether IL-37-MSCs could migrate to the injured intestine. The ileums were embedded in OCT to freeze the relevant section, and then, slides were observed under a fluorescent microscope to detect GFP expression.

### 2.8. Histology

To evaluate the impact of IL-37-MSC transplantation on the severity of intestinal IRI, the ileum was collected for assessment [27]. Intestine specimens were fixed in 10% formalin for 72 hours, then embedded in paraffin with correct orientation position of the crypt to villus axis and sectioned at 5 *μ*m for hematoxylin and eosin (H&E) staining to assess the severity of injury site. Chiu's score grading was used as standard: 0, normal villi of the small intestinal mucosa; 1, Gruenhagen's space under the intestinal mucosal epithelium in the villus axis, often accompanied by capillary congestion; 2, intestinal mucosal epithelium elevation from the intrinsic membrane and expansion of the intestinal subepithelial space; 3, large intestinal mucosal epithelium elevation, villus lodging to both sides, part of the villus shed; 4, villus and lamina detachment, bare capillaries dilate, an increase in the composition of lamina propria cell components; and 5, lamina propria is digested, bleeding or ulcers form [14, 28]. The slides from each sample and each slide with five fields at a magnification ×100 were observed by a professional pathologist, and the average scores of each group were calculated. The samples were randomly assigned to the pathologist and the experimental groups were blinded.

### 2.9. Enzyme-Linked Immunosorbent Assay (ELISA)

D-Lactic acid (D-Lac) and diamine oxidase (DAO) in the serum were used to assess the gut barrier function [29]. IL-1*β* in the tissue and serum was used to assess the local and systemic inflammation activity. DAO, D-Lac ELISA kits (Senbeijia, Nanjing, China), and IL-1*β* ELISA kit (DAKEWE, Shenzhen, China) were used to evaluate the levels of local and systemic inflammation in the serum or tissue. All operations were conducted according to the manufacturer's recommended protocol.

### 2.10. Real-Time PCR

Total RNA was obtained from ileum tissue by using RNAprep Pure Tissue Kit (Tiangen, Beijing, China), and reverse transcription was performed by FastQuant RT Super Mix (Tiangen, Beijing, China); mRNA expression was quantified by 2x SYBR Green qPCR Master Mix (Bimake, Houston, TX, USA). In the reaction system, *β*-actin was used as an internal normalizing gene, and mRNA expression was analyzed by comparing cycles of threshold (Ct value) of 2^-*ΔΔ*ct^. The primer sequences used were as follows: *β*-actin forward: 5′-GTTG ACAT CCGT AAAG AC-3′, reverse: 5′-TGGA AGGT GGAC AGTG AG-3′; TNF-*α* forward: 5′-ACAC ACGA GACG CTGA AGTA-3′, reverse: 5′-GGAA CAGT CTGG GAAG TCT-3′; and IL-6 forward: 5′-CTCA TTCT GTCT CGAG CCCA-3′, reverse: 5′-CTGT GAAG TCTC CTCT CCGG-3′.

### 2.11. Western Blot

Ileum tissues were homogenized, and the resulting total protein was extracted by RIPA lysis mixed with PMSF (Solarbio, Beijing, China). Then, 50 *μ*g of protein per sample was subjected to 7.5%, 10%, or 15% sodium dodecyl sulfate polyacrylamide gel electrophoresis (SDS-PAGE, Solarbio, Beijing, China). After overnight incubation at 4°C with anti-NLRP3 antibody (dilution at 1 : 1000, Abcam, Cambridge, UK), anti-caspase-1 antibody (dilution at 1 : 300, Santa Cruz, Oregon, USA, SC-398715), anti-IL-1*β* antibody (dilution at 1 : 600, Bioss, Beijing, China), anti-IL-18 antibody (dilution at 1 : 600, Bioss, Beijing, China), anti-*β*-actin antibody (dilution at 1 : 2000, Servicebio, Wuhan, China), and anti-HSP-90 antibody (dilution at 1 : 1000, Santa Cruz, Oregon, USA), the membranes with blotted proteins were then incubated with HRP-conjugated goat anti-rabbit secondary antibody (dilution at 1 : 2000, CST, Boston, USA) or rat anti-mouse secondary antibody (dilution at 1 : 2000, Servicebio, Wuhan, China) for an hour at room temperature. After washing three times with TBST, the electrochemiluminescence solution (ECL, Millipore, Massachusetts, USA) was added to the membranes, and then, the membranes were exposed to the exposure machine (ChemiScope series, Clinx Science Instruments Co., Ltd), and the resulting images were recorded and analyzed.

### 2.12. Statistics

Data were expressed as mean ± SD, and the differences among multiple groups were analyzed using one-way analysis of variance (ANOVA) after the normality test and followed by post hoc analysis with the least significant difference (LSD) test. Throughout the text, figures, and legends, the following terminologies are used to denote statistical significance: ∗*p* < 0.05; ∗∗*p* < 0.01; and ∗∗∗*p* < 0.001.

## 3. Results

### 3.1. Characterization of MSCs and IL-37-MSCs

**Figure 1:**
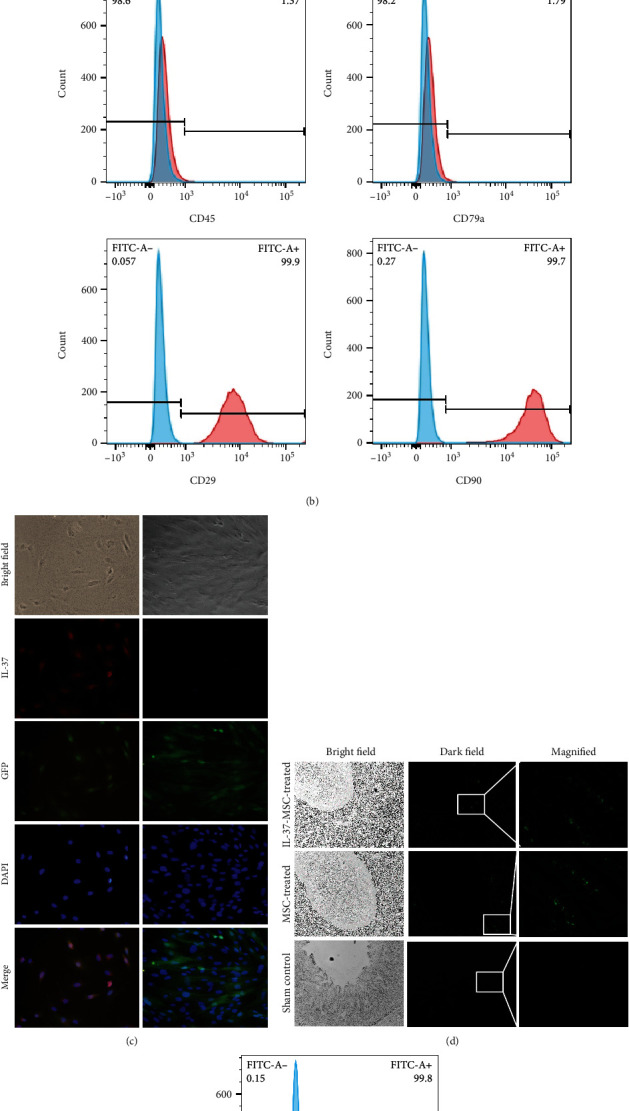
IL-37-MSCs and MSCs could migrate to the injured tissue. The morphology of MSCs. (a) Passages 0, 1, 2, and 3 of MSCs. (b) FACS analysis of MSC surface marker, surface expressions of CD29, CD45, CD79a, and CD90 were detected. (c) The IL-37 and GFP proteins were detected in IL-37-MSCs and MSCs. (d) IL-37-MSC GFP-positive rate was calculated; positive rate was above 99.8% which met our needs. (e) IL-37-MSC-treated and MSC-treated intestine exhibited significant GFP fluorescence while the sham group did not, which suggested that IL-37-MSCs and MSCs could migrate to the injured tissue.

After passage 2, MSCs showed spindle-shaped, fibroblast-like morphology and exhibited colony-forming abilities (Figure 1(a)). At passage 3, MSCs were detected and demonstrated high levels of expression of CD29 and CD90, but no expression of CD45 and CD79a (Figure 1(b)). At 72 hours posttransfection, the expression of GFP fluorescent protein was observed under a fluorescence microscope (Figure 1(c)). In addition, IL-37 expression was found in IL-37-MSCs but not in MSCs, as expected. After passing through the puromycin drug screening, the GFP-positive rate was above 99.8% measured by flow cytometry before cell treatment which met our needs (Figure 1(d)).

### 3.2. Transplanted IL-37-MSCs Could Migrate to the Injured Tissue *In Vivo*

To investigate whether IL-37-MSCs could migrate to the damaged intestine through the intravenous injection, intestine tissues were fixed by OCT to frozen section to detect the GFP expression. GFP expression was positive in the IL-37-MSC-treated and MSC-treated groups while the sham control group was negative, which meant that infused IL-37-MSCs and MSCs could migrate to the injured tissue (Figure 1(e)).

### 3.3. IL-37-MSCs Significantly Ameliorated Pathological IRI Damage of the Intestine

**Figure 2:**
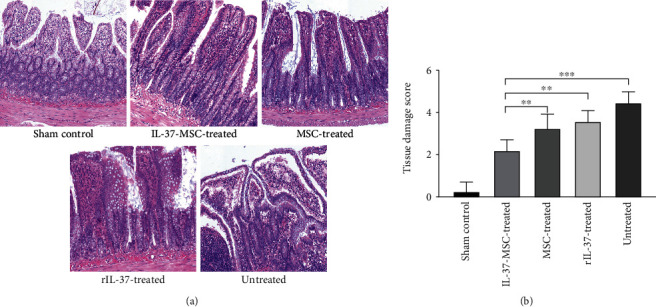
IL-37-MSCs significantly ameliorated pathological intestine damage following IRI. Microscopic findings illustrated the architecture of the ileum by 72 hours after reperfusion; the damage score was assessed according to Chiu's score. (a) Compared with the sham group, the untreated group demonstrated severe damage such as inflammatory cell infiltration, hemorrhage, and ulcer. However, as shown in (b), the IL-37-MSC-treated IRI group showed more significant therapeutic effects compared with the MSC-treated and rIL-37-treated groups. The data suggested that IL-37-MSCs provide a better protective role in intestinal IRI. Data shown were representative, and the *p* value was determined by one-way ANOVA followed by the LSD test. ∗*p* < 0.05, ∗∗*p* < 0.01, and ∗∗∗*p* < 0.001.

Chiu's score was used to assess the tissue damage. No obvious abnormal tissue changes were observed in the sham group (Figure 2(a)). However, intestinal injury in the untreated group was severe, which was characterized by villus damage, epithelial necrosis, subendothelial hemorrhage, and neutrophil infiltration. As expected, intestinal damage scores following IRI were significantly improved by the mere use of MSCs and rIL-37, furthermore improved by IL-37-MSC administration (IL-37-MSC treated *vs*. MSC treated, *p* < 0.01; IL-37-MSC treated *vs*. rIL-37 treated, *p* < 0.01, Figure 2(b)).

### 3.4. IL-37-MSCs Improved Intestinal Barrier Function following Intestinal IRI

**Figure 3:**
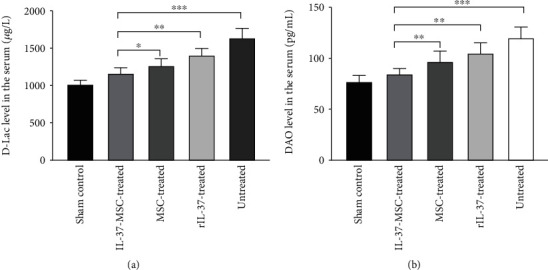
IL-37-MSCs could effectively improve intestinal barrier function following IRI. DAO and D-Lac were used to assess the gut barrier function. Serum samples were collected from the sham, IL-37-MSC-treated, MSC-treated, rIL-37-treated, and untreated IRI groups. In comparison with the MSC-treated and rIL-37-treated IRI groups, IL-37-MSCs significantly reduced serum levels of DAO and D-Lac, which meant IL-37-MSCs remarkedly improved gut barrier function. Data shown were representative, and the *p* value was determined by one-way ANOVA followed by the LSD test. ∗*p* < 0.05, ∗∗*p* < 0.01, and ∗∗∗*p* < 0.001.

To determine whether IL-37-MSCs could attenuate intestinal IRI, we measured the serum DAO and D-Lac, which represented the intestine barrier function as described above. As shown in Figure 3, it was found that DAO and D-Lac showed the highest levels in the untreated group, which reduced by different treatments. Compared with the MSCs- or rIL-37-treated group, IL-37-MSCs significantly decreased DAO and D-Lac levels in the serum (DAO level: IL-37-MSC treated *vs*. MSC treated, *p* < 0.01; IL-37-MSC treated *vs*. rIL-37 treated, *p* < 0.01, Figure 3(a); D-Lac level: IL-37-MSC treated *vs*. MSC treated, *p* < 0.05; IL-37-MSC treated *vs*. rIL-37 treated, *p* < 0.01, Figure 3(b)), which indicated that IL-37-MSCs could effectively improve intestine barrier function following intestinal IRI.

### 3.5. IL-37-MSCs Decreased Local and Systemic Inflammation Cytokine IL-1*β*

**Figure 4:**
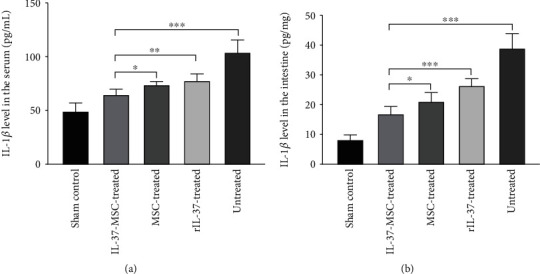
IL-37-MSCs could significantly decrease local and systemic inflammation activity. Local and systemic inflammation activity was assessed by IL-1*β* level in the local tissue and serum. Compared with MSC and rIL-37 treatment, IL-37-MSCs significantly alleviate the local and systemic inflammation activity. The *p* value was determined by one-way ANOVA followed by the LSD test. ∗*p* < 0.05, ∗∗*p* < 0.01, and ∗∗∗*p* < 0.001.

We used cytokine IL-1*β* level to evaluate local and systemic inflammation reactivity. Local and systemic cytokine IL-1*β* was significantly increased in the untreated IRI group (Figure 4). In addition, IL-37-MSC treatment significantly decreased local and systemic IL-1*β* level following IRI compared with MSCs and/or rIL-37 treatment (local IL-1*β* level: IL-37-MSC treated *vs*. MSC treated, *p* < 0.05; IL-37-MSC treated *vs*. rIL-37 treated, *p* < 0.001, Figure 4(a); systemic IL-1*β* level: IL-37-MSC treated *vs*. MSC treated, *p* < 0.05; IL-37-MSC treated *vs*. rIL-37 treated, *p* < 0.01, Figure 4(b)).

### 3.6. Infusion of IL-37-MSCs Decreased the NLRP3 Inflammasome Activation and Downstream Cascade Reactions

**Figure 5:**
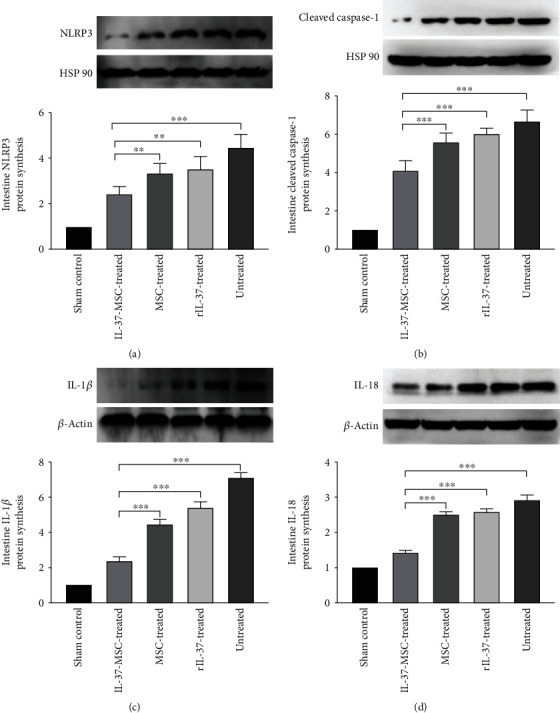
IL-37-MSC treatment decreased NLRP3 and downstream cascade protein synthesis. (a) Compared with MSC and IL-37 treatment, IL-37-MSCs significantly decreased the NLRP3 activation. In parallel with NLRP3 synthesis, cleaved caspase-1, IL-1*β*, and IL-18 proteins were significantly decreased following IL-37-MSC treatment compared with MSC and/or rIL-37 treatments (b–d), which suggested that IL-37-MSCs could inhibit the NLRP3-mediated signaling pathway. Data shown were representative, and the *p* value was determined by one-way ANOVA followed by the LSD test. ∗*p* < 0.05, ∗∗*p* < 0.01, and ∗∗∗*p* < 0.001.

NLRP3 played an important role in the development of various diseases, and inhibiting NLRP3 activation could effectively attenuate intestinal IRI. In this study, to detect whether IL-37-MSCs could effectively inhibit NLRP3 inflammasome activation, we performed western blot to detect NLRP3 and its downstream cascade reactions. As shown in Figure 5(a), NLRP3 synthesis in the IL-37-MSC-treated group was significantly lower than that in the MSC-treated and/or rIL-37-treated groups (IL-37-MSC treated *vs*. MSC treated, *p* < 0.01; IL-37-MSC treated *vs*. rIL-37 treated, *p* < 0.01). Cleaved caspase-1 proteins in each group were in accordance with NLRP3 synthesis; compared with sole MSC or rIL-37 treatment, IL-37-MSCs markedly decreased cleaved caspase-1 protein (IL-37-MSC treated *vs*. MSC treated, *p* < 0.001; IL-37-MSC treated *vs*. rIL-37 treated, *p* < 0.001, Figure 5(b)). As a result, mature form processing of IL-1*β* and IL-18 in tissues was also in accordance with NLRP3 synthesis; IL-1*β* and IL-18 proteins in the IL-37-MSC-treated group were lower than those in the MSC-treated or rIL-37-treated group (IL-1*β* protein: IL-37-MSC treated *vs*. MSC treated, *p* < 0.001; IL-37-MSC treated *vs*. rIL-37 treated, *p* < 0.001, Figure 5(c); IL-18 protein: IL-37-MSC treated *vs*. MSC treated, *p* < 0.001; IL-37-MSC treated *vs*. rIL-37 treated, *p* < 0.001, Figure 5(d)).

### 3.7. IL-37-MSC Treatment Decreased Local mRNA Expression for TNF-*α* and IL-6

**Figure 6:**
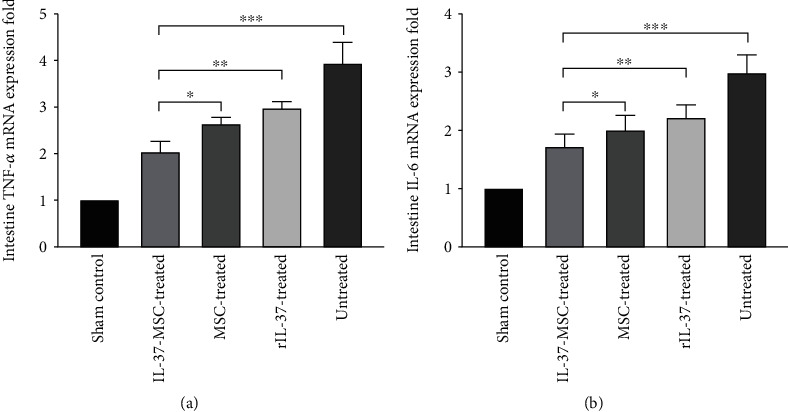
IL-37-MSC transplantation decreased IL-6 and TNF-*α* mRNA expression. IL-1*β* and IL-18 are key proinflammatory cytokines, and as their downstream proinflammatory molecules, IL-6 and TNF-*α* play a pivotal role in inflammatory reactivity. Intestine IL-6 and TNF-*α* mRNA expressions in the IL-37-MSC-treated group were significantly decreased compared with those in the MSC- and rIL-37-treated group. The *p* value was determined by one-way ANOVA followed by the LSD test. ∗*p* < 0.05, ∗∗*p* < 0.01, and ∗∗∗*p* < 0.001.

Proinflammatory cytokines count in the intestinal IRI. Hence, decreasing these proinflammatory cytokine expressions may contribute to alleviate tissue injury. As shown in Figure 6, the TNF-*α* and IL-6 mRNA expressions in the IL-37-MSC-treated group were obviously lower than those in the MSC-treated or rIL-37-treated IRI groups (TNF-*α* mRNA expression: IL-37-MSC treated *vs*. MSC treated, *p* < 0.05; IL-37-MSC treated *vs*. rIL-37 treated, *p* < 0.01, Figure 6(a); IL-6 mRNA expression: IL-37-MSC treated *vs*. MSC treated, *p* < 0.05; IL-37-MSC treated *vs*. rIL-37 treated, *p* < 0.01, Figure 6(b)).

## 4. Discussion

Our study, which investigated the therapeutic effects of IL-37-MSC treatment on inhibiting intestinal IRI, provided several preclinical implications of IL-37 and gene-modified MSCs. First, as compared with the sham control group, tissue damage scores (H&E assessment) were remarkably enhanced in animals with IRI. As expected, the parameter was significantly suppressed in animals with IRI after MSC or rIL-37 treatment and further notably decreased following IL-37-MSC transplantation therapy. Second, intestinal barrier function as measured by DAO and D-Lac was preserved in a manner consistent with tissue damage scores in all groups. Third, not only local but also systemic inflammatory cytokine IL-1*β* level was markedly attenuated in all treatment groups following IRI. Then, the NLRP3-mediated proinflammatory signaling pathway was found to be upregulated in the untreated IRI group, which was significantly suppressed following MSC, rIL-37, and IL-37-MSC treatments, suggesting the multifactorial nature of underlying mechanisms involved in intestinal IRI, for which IL-37-MSCs demonstrated much more powerful effects than MSCs or rIL-37 alone in attenuation of intestinal IRI.

The intestinal tract was one of the organs that were highly sensitive to ischemia. In addition, intestinal IRI usually leads to systemic inflammatory responses (SIRs) and multiple organ dysfunction syndromes (MODs), which is one of the highest morbidity and mortality diseases in the clinic [3]. Various inflammatory cells are involved in the whole process of disease, such as mucosal cells, macrophages, neutrophils, and endothelial cells, which are responsible for the release of different cytokines, chemokines, and free radicals following intestinal IRI. Therefore, we believe that the mechanism of ischemia reperfusion injury is closely related to the release of ROS after ischemia or hypoxia [7]. Excessive ROS activated NLRP3 inflammasome and eventually led to the excessive release of proinflammatory cytokines IL-1*β* and IL-18 [7]. The dysregulation of NLRP3 inflammasome could cause excessive inflammation and play a pivotal role in many human diseases. Previous studies have demonstrated that NLRP3 inflammasome is involved in ischemia reperfusion injury in several organs such as the heart, liver, and kidney. Interestingly, little has been reported about the role of NLRP3 in intestinal ischemia reperfusion injury until Wang et al. identified that NLRP3 actually count in intestinal IRI [30]. Several studies suggested that targeting inhibiting NLRP3 inflammasome activation could alleviate ischemia reperfusion injury occurring in different organs [7, 12, 13, 31, 32]. Therefore, inhibition of NLRP3 activation is incredibly effective at treating intestinal IRI.

A broad base of research supports cell therapy strategies to be a strongly effective way in the treatment of different diseases [16, 18, 33, 34]. MSCs act as adult stem/stromal cells that could differentiate into specific tissues cells induced by local microenvironment when they were damaged and could secrete various cytokines. Previous studies have demonstrated that MSCs, especially those adipose derived, possess anti-inflammatory and immunomodulatory functions. Intriguingly, MSCs and IL-37 both could inhibit NLRP3 inflammasome activation, but research has suggested different mechanisms were involved. MSCs have been noted for ischemia reperfusion injury, and good results have been obtained, which could decrease NLRP3 activation via clearing excessive ROS as reported [18]. However, as described above, IL-37 plays an important anti-inflammatory and immunomodulatory capacity in a variety of inflammatory and autoimmune diseases. In addition, IL-37 *in vivo* could inhibit NLRP3 activation also in colitis- and LPS-induced disease [19, 22, 35–37]. In a recent study, Rudloff et al. reported that IL-37 significantly suppresses inflammasome activity *in vivo* to ameliorate inflammasome-driven diseases, which could corroborate our study results to some extent [38].

However, either mesenchymal stromal cells or IL-37 showed limited roles to relieve intestinal IRI [25, 26, 39]. Thus, it was very important to find a way to enhance the effects of MSCs. During the past decades, remarkable progresses have been made in the area of gene-engineered MSC-based therapy. Based on these points, we utilized MSCs as a vehicle to drive IL-37 and further release IL-37 *in vivo* (the result is demonstrated in the supplementary figure (available here) that IL-37 expression in the serum in the IL-37-MSC-treated group was higher than that in the MSC-treated group) to help better inhibit NLRP3 inflammasome activation to alleviate damage, thereby enhancing the effects of MSCs in the intestinal IRI model.

This is the first study to demonstrate that IL-37 could play a protective role in the intestinal IRI and illustrated that IL-37 gene modification could enhance the therapeutic effects of MSCs in the ischemia reperfusion injury. However, many mechanisms are involved in the IL-37-MSC treatment in intestinal IRI, and more specific mechanisms remain to be explored.

## 5. Conclusion

In conclusion, IL-37 gene modification could enhance the therapeutic effects of MSCs. IL-37-MSCs improved intestine barrier function, improved injured tissue microenvironment, and inhibited the NLRP3-mediated signaling pathway, which exert a much better protective role in intestinal IRI than MSCs. NLRP3-related signaling pathways could be related to the process of IL-37-MSC-mediated protection. IL-37-MSC treatment acted as an effective tool to protect the intestine against IRI.

## Data Availability

The dataset supporting the conclusions of this article is included within the article.

## Abbreviations

MSCs: Mesenchymal stromal cells
rIL-37: Recombinant interleukin 37
IL-37-MSCs: Interleukin 37 gene-modified mesenchymal stromal/stem cells
IRI: Ischemia reperfusion injury
SMA: Superior mesenteric artery
NOD-like: Nucleotide-binding oligomerization domain-like
NLRP3: NOD-like receptor family, pyrin domain protein 3
FBS: Fetal bovine serum
PBS: Phosphate buffer saline
H&E: Hematoxylin and eosin
DAO: Diamine oxidase
D-Lac: D-Lactate
IL-1*β*: Interleukin 1*β*
IL-18: Interleukin 18.

## Supplementary Materials

Supplementary Materials Supplementary figure: IL-37-MSC treatment increased IL-37 level in the serum. As shown in the supplementary figure, IL-37 level in the IL-37-MSC-treated group was higher than that in the MSC-treated group. The *p* value was determined by one-way ANOVA followed by the LSD test. ∗*p* < 0.05, ∗∗*p* < 0.01, and ∗∗∗*p* < 0.001.
