# Supplementary figures and images for: IL-37 Gene Modification Enhances the Protective Effects of Mesenchymal Stromal Cells on Intestinal Ischemia Reperfusion Injury
Source: Stem Cells Int. 2020 Aug 7;2020:8883636. doi: 10.1155/2020/8883636 (PMC7439787; doi:10.1155/2020/8883636)

Supplementary figure

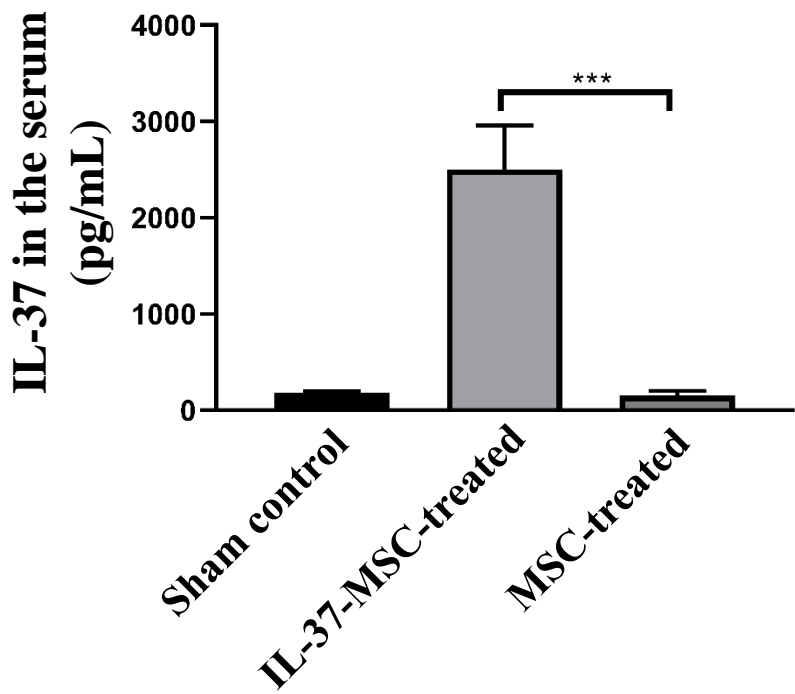

Supplement: Supplementary Materials — Supplementary figure: IL-37-MSC treatment increased IL-37 level in the serum. As shown in the supplementary figure, IL-37 level in the IL-37-MSC-treated group was higher than that in the MSC-treated group. The p value was determined by one-way ANOVA followed by the LSD test. ∗p < 0.05, ∗∗p < 0.01, and ∗∗∗p < 0.001. [file 8883636.f1.pdf]
